
JOURNAL INFORMATION
==============================
NLM Title Abbreviation: ScientificWorldJournal
Journal ID: ScientificWorldJournal
NLM Journal ID: 101131163
Journal ID: TSWJ

The Scientific World Journal

ISSN: 2356-6140
EISSN: 1537-744X
Publisher: Wiley

ARTICLE INFORMATION
==============================
PMCID: PMC6009521
PMID: 29973797
DOI: 10.1100/tsw.2002.23
Article ID: 647129
Article version: 1
Subjects: Short Report

Studying Protein-Excited States by Nmr

Mittermaier Tony
Mulder Frans
Dahlquist Rick
Kay Lewis E. *
Departments of Medical Genetics, Biochemistry and Chemistry, University of Toronto, Toronto, Ontario, Canada, M5S 1A8 and Department of Chemistry, University of Oregon, Eugene, Oregon 97403, USA
*Lewis E. Kay: kay@pound.med.utoronto.ca
Publication date: 2002
Electronic publication date: 2002 Jun 25
Volume: 2
First page: 45
Last page: 46
Copyright: Copyright © 2002 Tony Mittermaier et al.
Copyright year: 2002
License: This is an open access article distributed under the Creative Commons Attribution License, which permits unrestricted use, distribution, and reproduction in any medium, provided the original work is properly cited.
License URL: https://creativecommons.org/licenses/by/3.0/

==============================
Advances in Gene Technology: The Genome and Beyond –
Structural Biology for Medicine (Proceedings of the 2002 Miami
*Nature Biotechnology* Winter Symposium)
*TheScientificWorld* 2002, 2(S2), 45–46
ISSN 1532-2246; DOI 10.1100/tsw.2002.23

### STUDYING PROTEIN-EXCITED STATES BY NMR

Tony Mittermaier, Frans Mulder, Rick Dahlquist, and Lewis E. Kay
Departments of Medical Genetics, Biochemistry and Chemistry, University of Toronto, Toronto, Ontario, Canada, M5S 1A8 and Department of Chemistry, University of Oregon, Eugene, Oregon 97403
kay@pound.med.utoronto.ca

**INTRODUCTION**. Much of structural biology has focused on obtaining static three-dimensional representations of molecules and molecular complexes. However, a complete description of a protein requires not only a three-dimensional picture of the average structure but knowledge of how that structure changes with time. Proteins are inherently dynamic and the dynamics are critical for function. For example, it is often the case that static three-dimensional structures alone do not completely explain results from functional biological assays, nor do they necessarily illuminate the path for protein engineering or rational drug design. This is, of course, not surprising. A three-dimensional static structure provides a description of the ground state of a molecule. Macromolecular function is, in many cases, highly dependent on excursions to excited molecular states and hence intimately coupled to flexibility. For example, X-ray studies of a cavity mutant of T4 lysozyme, L99A, show that the cavity is sterically inaccessible to ligand, yet the protein is able to bind substituted benzenes rapidly. We have used novel relaxation dispersion NMR methods to characterize the transition between a ligand-inaccessible closed form of the protein and an excited state that binds ligands that is 2.0 kcal $mol^{-1}$ higher in free energy.

**METHOD**. Conformational exchange on a μs-ms time scale leads to a modulation of the chemical shift of an NMR active nucleus, resulting in a contribution to the effective decay of transverse signal termed $R_{ex}$.

This contribution is a direct result of the chemical shift difference between the spin in each of the exchanging conformations and can be suppressed by the application of radio frequency $B_1$ fields. Thus, the effective decay rate of magnetization, $R_2^{eff}$, decreases as a function of increasing $B_1$ field strength, $\nu_{CPMG}$. Relaxation dispersion profiles, $R_2^{eff}$ *vs* $\nu_{CPMG}$, can be measured and subsequently analyzed to obtain differences in chemical shifts between exchanging sites, as well as kinetic (rates of interconversion) and thermodynamic (populations) parameters describing the exchange process.

**RESULTS**. The relaxation data on the protein are well fit by a model in which L99A undergoes a conformational transition from a highly populated (97%, 25°C) ground state to an excited conformation, which is 2.0 kcal/mol higher in free energy. A temperature-dependent study of the rates of interconversion between ground and excited states shows that ΔH = 7.1 kcal/mol and TΔS

= 5.1 kcal/mol for this process at 25$^{o}$C. The residues involved cluster about the cavity, providing compelling evidence that the excited state facilitates ligand entry.

**DISCUSSION.** NMR data establishes that the ground to excited state transition involves a conformational rearrangement that includes the C-terminal portion of helix E, helix F and helix I and the loops that connect these elements to the protein. These experiments are quite remarkable in that detailed kinetic, thermodynamic, and structural information is available about the excited state despite the fact that its population is too low to be observed directly in even the most sensitive of NMR spectra. The methods developed can be applied to a variety of biological systems and used to provide a quantitative kinetic and thermodynamic basis for understanding how ligands enter occluded binding sites and how populations of excited states might be involved in the process.

**REFERENCES**

1. Mulder, F.A., Hon, B., Muhandiram, D.R., Dahlquist, F.W., and Kay, L.E. (2000) Biochemistry 39, 12614–12622.
2. Skrynnikov, N.R., Mulder, F.A.A., Hon, B., Dahlquist, F.W., and Kay, L.E. (2001) J. Am. Chem. Soc. 123, 4556–4566.
3. Mulder, F.A.A., Mittermaier, A., Hon, B., Dahlquist, F.W., and Kay, L.E. (2001) Nat. Struct. Biol., in press.
